# Autonomic Nerve Fiber Compositions of Zygomaticotemporal and Lacrimal Nerves: A Cadaveric Immunohistochemical Analysis

**DOI:** 10.1002/alr.70234

**Published:** 2026-08-09

**Authors:** Bushra Rahman, Hangil Lee, Kelly A. Shaftel, Abdurrahman Abdurrob, Yusra Mansour, Swapna Vemuri, Jun Jin, Jack P. Rock, John R. Craig

**Affiliations:** ^1^ Department of Ophthalmology Henry Ford Health Detroit Michigan USA; ^2^ Department of Neurosurgery Henry Ford Health Detroit Michigan USA; ^3^ Department of Otolaryngology‐Head and Neck Surgery Henry Ford Health Detroit Michigan USA; ^4^ Department of Public Health Sciences Henry Ford Health Detroit Michigan USA; ^5^ Department of Otolaryngology‐Head and Neck Surgery Michigan State University College of Human Medicine Detroit Michigan USA

## Abstract

- Zygomaticotemporal (ZTN) and lacrimal nerves (LN) both carried sympathetics and parasympathetics
- ZTNs had more parasympathetics than LNs, but markers varied significantly within and between nerves
- LN parasympathetics to the lacrimal gland may mitigate dry eye after vidian neurectomy

## 1 Introduction

The lacrimal gland (LG) is a tubuloacinar exocrine gland in the superolateral orbit that produces the majority of the ocular tear film, and it is modulated by both peripheral sensory and autonomic innervation and central nervous pathways [1]. Parasympathetic innervation is largely responsible for stimulating LG acinar cells to secrete tear fluid [2, 3]. The vidian nerve (VN) has historically been reported to be the main parasympathetic supply to the LG by way of postsynaptic fibers from sphenopalatine ganglion (SPG) traveling in the zygomaticotemporal nerve (ZTN), multiple individual rami, or by lacrimal nerve (LN) communication [4, 5]. In addition, the VN also provides parasympathetic innervation to nasal submucosal glands by way of posterior nasal nerves (PNNs) [6, 7].

Following an endoscopic vidian neurectomy (EVN), patients often experience reduced rhinorrhea and tearing, but they rarely develop significant xerophthalmia [8, 9, 10]. While this implies that the LG must receive additional parasympathetic innervation, such alternative sources have not been well‐established. This study aimed to compare sympathetic and parasympathetic nerve fiber compositions between LNs and ZTNs to explore whether the LN is an alternative source of LG autonomic innervation.

## 2 Methods

Nine fresh cadaver heads were dissected to harvest approximately 1 cm of LN and ZTN sections from one orbit per cadaver. The LN was sectioned at its proximal‐most entry into the orbital apex through the superior orbital fissure, and the ZTN was sectioned at its orbital entry through the inferior orbital fissure (Figure 1). Specimens were formalin‐fixed, sectioned, and stained with choline acetyltransferase (ChAT) and tyrosine hydroxylase (TH) antibodies, and then with fluorophores to selectively identify ChAT (Opal 520, green) and TH (Opal 620, yellow); see the methods in File S1. Positive and negative controls were used to establish marker thresholds. Areas of positive marker staining were measured and expressed as percentages of total tissue area using Visiopharm software (Horsholm, Denmark). ChAT and TH percent areas (PAs) and coefficients of variance (CV) were compared within and between nerve specimens using paired Student's *t*‐tests for mean marker PAs and paired label‐swap permutation tests for marker variances. *p*‐values ≤ 0.05 were considered statistically significant.

**FIGURE 1:**
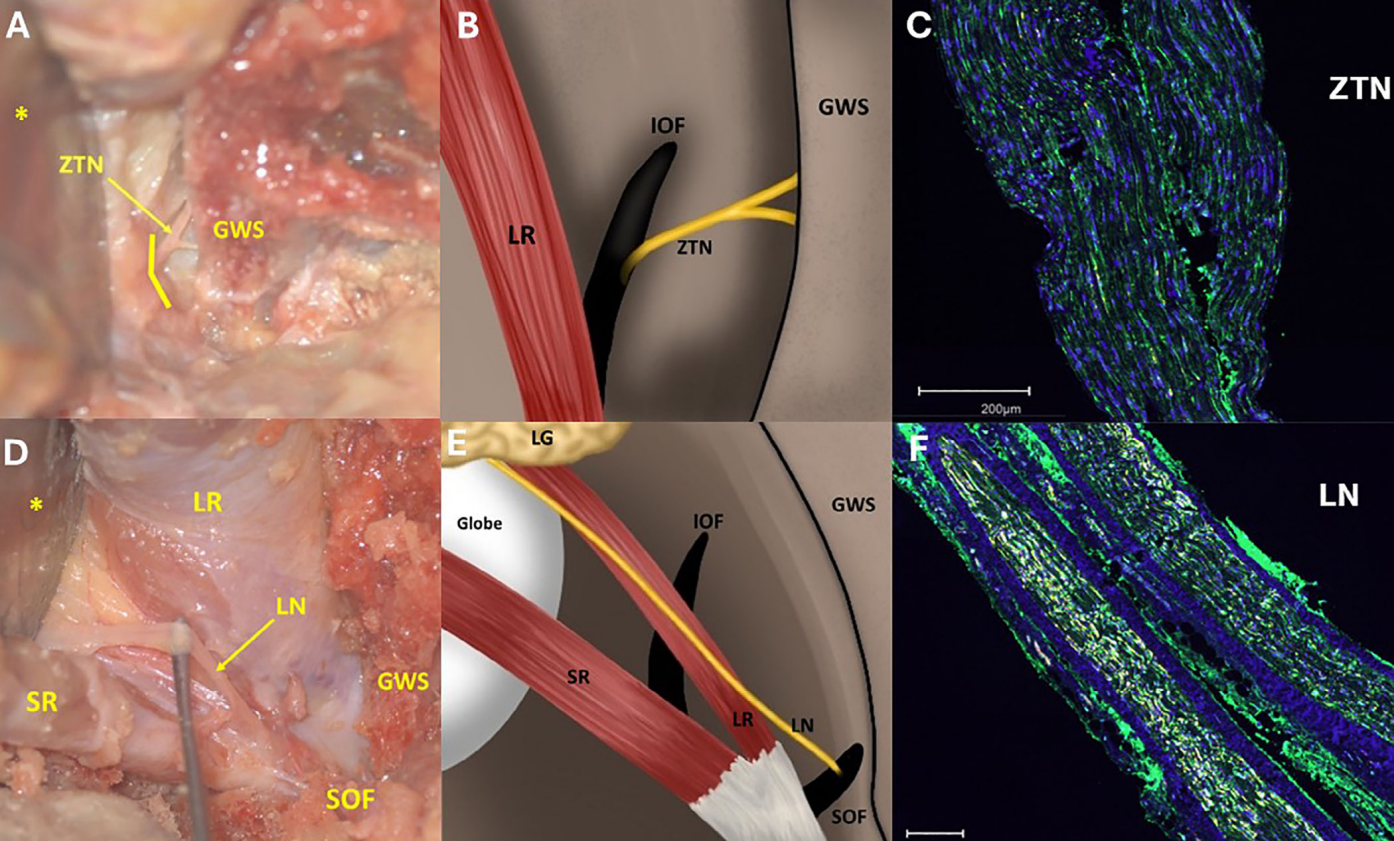
Gross anatomic dissection of one cadaver's left orbital apex, and the immunohistochemical and fluorescent staining of the zygomaticotemporal (ZTN) and lacrimal nerves (LN) in the same cadaver. (A) Dissection showing the ZTN emanating from the inferior orbital fissure (yellow line), coursing along the orbital floor and greater wing of the sphenoid (GWS) bone (which was cut down with drill and Kerrison rongeurs). Orbital fat and extraocular muscles were retracted with a malleable retractor (yellow asterisk). (B) Diagram illustrating the correct anatomic relationships from (A) involving the lateral rectus muscle (LR), without distortion from a retractor. It shows the cut edge of the GWS as the posterior boundary of the orbital apex. (C) Immunofluorescent staining of the ZTN from (A), showing a greater percent area of choline acetyltransferase (ChAT; green) compared to tyrosine hydroxylase (TH; yellow). (D) Dissection showing the LN coursing along the superior rectus muscle (SR) after emanating from the superior orbital fissure (SOF). Note that the malleable retractor (yellow asterisk) distorted the relationship among the LN, superior rectus muscle (SR), and lateral rectus muscle (LR). (E) Diagram illustrating the correct anatomic relationships from (D) between the LN, SOF, LR, superior rectus muscle (SR), and lacrimal gland (LG), without distortion from a retractor. Notably, the LN courses over the superior aspect of the LR, but had been pulled medially by the retractor in (D). (F) Immunofluorescent staining of the LN from (C), again showing a greater percent area of choline acetyltransferase (ChAT; green) compared to tyrosine hydroxylase (TH; yellow), though there was more TH staining in this specimen than in the ZTN in (B).

## 3 Results

Of the 9 cadavers, mean age was 78.2 ± 10.0 years, 55.6% were male, 8/9 were White, and one was Black. Table 1 shows the results of different ChAT and TH comparisons between and within nerves. ChAT PA was greater in ZTNs compared to LNs (24.6% vs. 14.1%, *p* = 0.004), while TH PA was equivalent between ZTNs and LNs (3.7% vs. 3.8%, *p* = 0.884). Within each nerve, ChAT PA was greater than TH PA (ZTN: 24.6% vs. 3.7%, *p* = 0.0003; LN: 14.1% vs. 3.8%, *p* = 0.026). Both ChAT and TH PAs demonstrated high CVs across LNs and ZTNs (CV > 0.3). ChAT and TH CVs were equivalent between LNs and ZTNs (*p* > 0.121) and were not significantly different when compared to one another within each nerve (*p* > 0.172).

**TABLE 1:** Comparisons of choline acetyltransferase (ChAT) and tyrosine hydroxylase (TH) markers between and within zygomaticotemporal (ZTN) and lacrimal nerves (LN).

Each marker compared between nerves	ZTN	LN	*p*‐value
ChAT % area [mean (SD)]	24.6 (10.3)	14.1 (14.2)	**0.004**
TH % area [mean (SD)]	3.7 (3.6)	3.8 (2.5)	0.884
ChAT CV	0.420	1.010	0.121
TH CV	0.982	0.645	0.383

Markers compared within nerves	ChAT	TH	*p*‐value
Marker % area in ZTN [mean (SD)]	24.6 (10.3)	3.7 (3.6)	**0.0003**
Marker % area in LN [mean (SD)]	14.1 (14.2)	3.8 (2.5)	**0.026**
CV in ZTNs	0.420	0.982	0.172
CV in LNs	1.010	0.645	0.547

*Note*: Bolded *p*‐values were statistically significant.

Abbreviation: CV, coefficient of variance.

## 4 Discussion

The VN has been considered the predominant autonomic supply to the LG by way of the ZTN or other postganglionic SPG fibers [2, 4, 5]. Accordingly, patients who undergo EVN during endoscopic skull base or rhinologic surgeries often experience decreased tearing. However, patients rarely develop significant xerophthalmia [8, 9]. Authors have considered increased basal tearing or reinnervation as possible mechanisms behind the lack of dry eye following EVN [8]. However, an alternative parasympathetic nerve supply could also explain this, but has not been established. Historically, the ZTN reportedly sent communicating branches to the LN before supplying the LG, implying that any LG autonomic supply originates from the VN [11]. More recently, Scott et al. showed in a cadaver study that 60% of specimens demonstrated ZTNs and LNs supplying the LG separately without communicating branches between them [4]. While they showed that the LN and ZTN can individually innervate the LG, they did not assess immunohistochemically assess for autonomic fibers within the nerves.

The current study demonstrated immunohistochemically that both the ZTN and LN contained parasympathetic fibers, with the ZTN containing a significantly greater proportion of parasympathetic fibers than the LN. This not only further reinforces that the VN is the predominant supply of the LG's parasympathetic innervation, but also highlights importantly that the LN is at least one other source of LG parasympathetic innervation. In addition, both nerves contained significantly greater parasympathetic than sympathetic nerve fiber density. This is in line with prior studies reporting that the LG is preferentially parasympathetically innervated. Another interesting finding was that each autonomic marker varied substantially within ZTNs and LNs. This could explain variable initial and long‐term effects on lacrimation in patients who undergo EVN. For example, patients with greater LN parasympathetic supply may be more resistant to developing postoperative xerophthalmia following EVN.

Multiple studies have explored the effects of EVN on lacrimation. Wang et al. showed in a prospective study of 21 EVN patients that tearing was significantly reduced by 24% at 1 month postoperatively in EVN eyes, while control eyes showed no changes. Tearing reduction persisted at 3 months postoperatively (90% of original tear secretion), but was not significantly different from preoperative levels [8]. Other series have also demonstrated decreased tearing initially after EVN, with tearing returning to normal or near‐normal by 3–6 months postoperatively [9, 12, 13]. Based on the current study, it is possible that the LN provides a compensatory increase in LG parasympathetic innervation following EVN.

Another implication of this study's findings pertains to PNN treatments for chronic rhinitis, including both ablations and neurectomies. One of the benefits of treating the PNNs over the VN is that xerophthalmia should be exceedingly rare [7]. The VN carries preganglionic autonomic fibers to the SPG. These fibers synapse onto separate ganglionic cell populations, which send postganglionic fibers to the nasal mucosa via PNNs, and to the LG via the ZTN (Figure 2). While PNN treatments should not lead to xerophthalmia, there have been rare reports of this [7]. One theoretical mechanism behind this could be that PNN treatments cause ZTN ganglionic cell damage, but this would be theoretical and requires further study.

**FIGURE 2:**
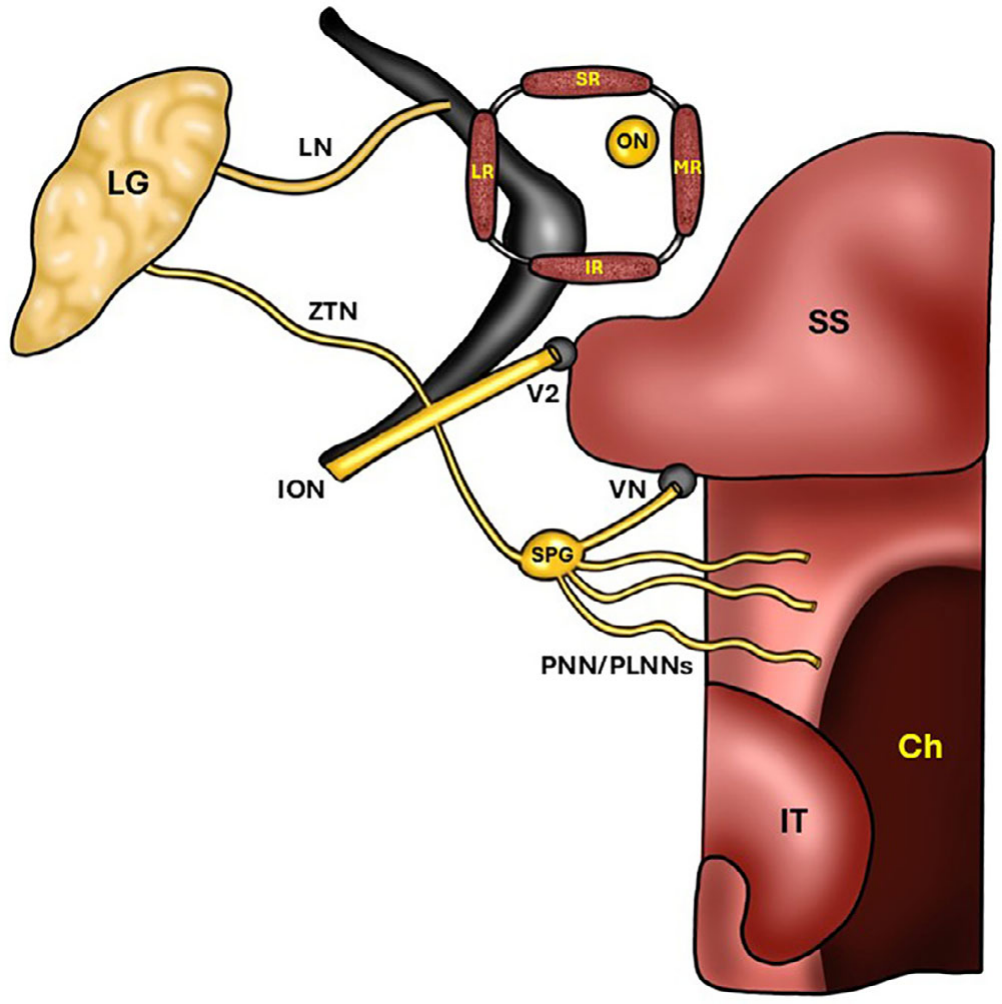
Diagram illustrating innervation pathways to the lacrimal gland (LG) from the vidian nerve (VN) and trigeminal nerve branches (V1 by lacrimal nerve [LN], and V2 by zygomaticotemporal nerve [ZTN]). The VN provides preganglionic autonomic nerve fibers to the sphenopalatine ganglion (SPG) in the pterygopalatine fossa. Postganglionic fibers then supply the posterior nasal cavity via the posterior and posterolateral nasal nerves (PNNs, PLNNs), and the LG by separate fibers joining the ZTN (V2). Separately, the LN (V1) emanates from the superior orbital fissure (SOF) to supply the LG. Ch, choana; IOF, inferior orbital fissure; IR, inferior rectus muscle; IT, inferior turbinate; LR, lateral rectus muscle; MR, medial rectus muscle; ON, optic nerve; SR, superior rectus muscle; SS, sphenoid sinus.

Main study limitations were the small cadaveric sample size limiting generalizability, and potential limitations of immunohistochemical staining and analysis. In addition, since systemic and ophthalmologic disease states in cadavers were unknown, whether these could have affected ChAT and TH PAs and variances cannot be determined. A final limitation was that the origin of LN cholinergic fibers could not be determined. However, one important point to highlight was that parasympathetic fibers in the LN could not have originated from the ZTN or other postganglionic fibers from the SPG, since LN specimens were harvested from the LN's proximal‐most entry into the orbital apex, proximal to the ZTN's entrance into the orbit. It is possible that the greater superficial petrosal nerve sends branches to the LN, but whether separate pathways exist between facial nuclei and the trigeminal system will require further study.

In conclusion, this study showed that while the ZTN provided the majority of the LG's parasympathetic supply (by way of VN origin), the LN provided at least one additional source of parasympathetic innervation. This could help explain why most patients do not develop clinically significant xerophthalmia following EVN.

## Funding

The authors have nothing to report.

## Conflicts of Interest

John R. Craig is a consultant for Aerin Medical, Inc. The other authors declare no conflicts of interest.

## Supporting information




**Supplementary File 1**: alr70234‐sup‐0001‐SuppMat.docx
