# Supplementary material for: Autonomic Nerve Fiber Compositions of Zygomaticotemporal and Lacrimal Nerves: A Cadaveric Immunohistochemical Analysis
Source: Int Forum Allergy Rhinol. 2026 Aug 9;16(10):1202–5. doi: 10.1002/alr.70234 (PMC13634543; doi:10.1002/alr.70234)
Supplement: Supplementary file 1 [file ALR-16-1202-s001.docx]

**Supplemental File 1**

Staining Method:

Opal assay procedure- Fluorophores were assigned to markers of interest.

After sectioning nerve specimens, study sample slides and positive/ negative control tissue slides were labeled with all markers of interest plus DAPI nuclear stain. (Chat-1:500 dilution-Opal 520 FITC-Green and TH-1:250 dilution-Opal 620 Texas Red).

Slides were prepped following the steps below-

Antigen Retrieval Steps

a. HIER – Biocare Decloaker
b. Program 4 (95˚C) for 20 minutes
c. Fill one container with required AR 9 solution; Others with 250 mL DiH2O
d. Put slides in solution before heating
e. When the Decloaker is finished after 20 minutes at 95˚C, remove the lid and remove the inner pot of the pressure cooker and let it cool on the counter for 15 minutes.
f. DiH2O rinse
g. Draw around the tissue with a PAP Pen
h. TBST rinse
i. Prepare the antibody in the RTU Opal Antibody Diluent/Block Solution

Blocking-Use the Ready-to-Use Opal Antibody Diluent/Block Solution for 10 minutes at RT in a humidity chamber. Blocker was removed, incubate primary antibody for 30 minutes, rinse with TBST, secondary antibody-working HRP solution was incubated for 10 minutes and rinsed with TBST. Fluorophore incubation was performed Opal 520 FITC. Next, antibody stripping was performed by the Biocare decloaker, add the next primary antibody TH 1:250 for 30 minutes, rinse, secondary antibody incubation was performed from the working HRP solution, rinse, and fluorophore incubation Opal 620 Texas Red. Last, DAPI incubation was performed for 5 minutes and slides were cover-slipped with prolong diamond mounting media. Let slides air dry in the dark and store in 4˚C.
